# DNMT3A R882H mutation drives daunorubicin resistance in acute myeloid leukemia via regulating NRF2/NQO1 pathway

**DOI:** 10.1186/s12964-022-00978-1

**Published:** 2022-10-27

**Authors:** Xuan Chu, Liang Zhong, Wenran Dan, Xiao Wang, Zhonghui Zhang, Zhenyan Liu, Yang Lu, Xin Shao, Ziwei Zhou, Shuyu Chen, Beizhong Liu

**Affiliations:** 1 Central Laboratory of Yong-Chuan Hospital, Chongqing Medical University, Chongqing, 402160 China; 2 Key Laboratory of Laboratory Medical Diagnostics, Ministry of Education, Department of Laboratory Medicine, Chongqing Medical University, Chongqing, 400016 China

**Keywords:** AML, DNMT3A R882H mutation, Daunorubicin, Chemoresistance, NRF2

## Abstract

**Background:**

DNA methyltransferase 3A (DNMT3A) often mutate on arginine 882 (DNMT3A^R882^) in acute myeloid leukemia (AML). AML patients with DNMT3A R882 mutation are usually resistant to daunorubicin treatment; however, the associated mechanism is still unclear. Therefore, it is urgent to investigate daunorubicin resistance in AML patients with DNMT3A R882 mutant.

**Method:**

AML cell lines with DNMT3A-wild type (DNMT3A-WT), and DNMT3A-Arg882His (DNMT3A-R882H) mutation were constructed to investigate the role of DNMT3A R882H mutation on cell proliferation, apoptosis and cells’ sensitivity to Danunorubin. Bioinformatics was used to analyze the role of nuclear factor-E2-related factor (NRF2) in AML patients with DNMT3A R882 mutation. The regulatory mechanism of DNMT3A R882H mutation on NRF2 was studied by Bisulfite Sequencing and CO-IP. NRF2 inhibitor Brusatol (Bru) was used to explore the role of NRF2 in  AML cells carried DNMT3A R882H mutation.

**Results:**

AML cells with a DNMT3A R882H mutation showed high proliferative and anti-apoptotic activities. In addition, mutant cells were less sensitive to daunorubicin and had a higher NRF2 expression compared with those in WT cells. Furthermore, the NRF2/NQO1 pathway was activated in mutant cells in response to daunorubicin treatment. DNMT3A R882H mutation regulated the expression of NRF2 via influencing protein stability rather than decreasing methylation of NRF2 promoter. Also, NRF2/NQO1 pathway inhibition improved mutant cells’ sensitivity to daunorubicin significantly.

**Conclusion:**

Our findings identified NRF2 as an important player in the regulation of cell apoptosis through which helps mediate chemoresistance to daunorubicin in AML cells with DNMT3A R882H mutation. Targeting NRF2 might be a novel therapeutic approach to treat AML patients with a DNMT3A R882H mutation.

**Video abstract**

**Supplementary Information:**

The online version contains supplementary material available at 10.1186/s12964-022-00978-1.

## Introduction

Acute myeloid leukemia (AML) is a type of malignant blood disease characterized by a rapid development and treatment inefficiency, recurrence and multidrug resistance in AML still remain to be resolved [1]. The tumor suppressor, DNA methyltransferase 3A (DNMT3A) [2], is involved in regulating DNA methylation. Studies have confirmed that about 20% AML patients are accompanied with DNMT3A mutations [3]. DNMT3A mutations contain several point mutations, R882H and R882C mutations being the most common mutations [4]. Recent studies identified that DNMT3A R882 mutation is associated with reoccurrence, incomplete response rate, and low average survival time in AML patients. This makes DNMT3A R882 mutation a dependent prognosis factor in AML patients [5–8].

Till now, researchers have explored xenogeneic hematopoietic stem cell transplantation [9] and chemotherapy [10, 11] in the treatment of AML patients with DNMT3A R882 mutation. Some studies suggested that only a high concentration of daunorubicin can obviously increase the survival rate for those patients [12] and promote cell apoptosis in AML cells with DNMT3A R882H mutation [13]. These observations indicated that DNMT3A R882H mutation may mediate drug resistance to daunorubicin. Despite the effective therapeutic effect of high concentration of daunorubicin, its side effects should not be ignored. Studies have shown that high concentration of daunorubicin often causes adverse reactions such as infection, hemorrhage, suppression of bone marrow function, and cardiac toxicity [14]. Therefore, it is important to investigate the resistance mechanism of mutant cells to daunorubicin to improve its curative effect as well as reduce the adverse drug reaction and toxic effect.

Nuclear factor-E2-related factor (NRF2) is an important transcription factor which can be activated by drug treatment, toxic substances, carcinogens, and oxidative stress [15, 16] to prevent chemical and radiation induced tumorigenesis [17–20]. However, the hyperactivation of the NRF2 pathway can protect cancer cells from oxidative stress, chemotherapeutic agents, and radiotherapy. These events were observed in different types of cancer such as breast cancer, lung cancer, colon cancer, and pancreatic cancer [21–24]. Brusatol, which is extracted from Brucea javanica, is a unique inhibitor of NRF2 through reducing its expression [25]. Previous studies have shown that brusatol has great effects on different cancers [26–28] and that NRF2 could mediate drug resistance in AML cells [29–31]. Therefore, we hypothesized that NRF2 may play a role in mediating drug resistance to daunorubicin in AML with DNMT3A R882H mutation.

Experimentally, our results demonstrated that DNMT3A R882H obviously increases tumor cells’ proliferation and restricts cell apoptosis. In addition, mutant cells showed a low sensitivity to daunorubicin treatment compared with that in wild-type cells. After investigating the mechanism, we found that the expression of NRF2 and its downstream NQO1 (NAD (P) H: quinone oxidoreductase 1) were significantly increased in mutant cells. Moreover, DNMT3A R882H mutation may affect the sensitivity of the AML cells to daunorubicin via activating the NRF2 /NQO1 pathway.

Collectively, we identified that NRF2 mediates drug resistance to daunorubicin by regulating cell growth and apoptosis in AML cells with DNMT3A R882H mutation. Thus, the inhibition of NRF2 may have a guiding significance for the treatment of AML patients.

## Results

### DNMT3A-R882H influences cell proliferation and apoptosis

**Fig. 1:**
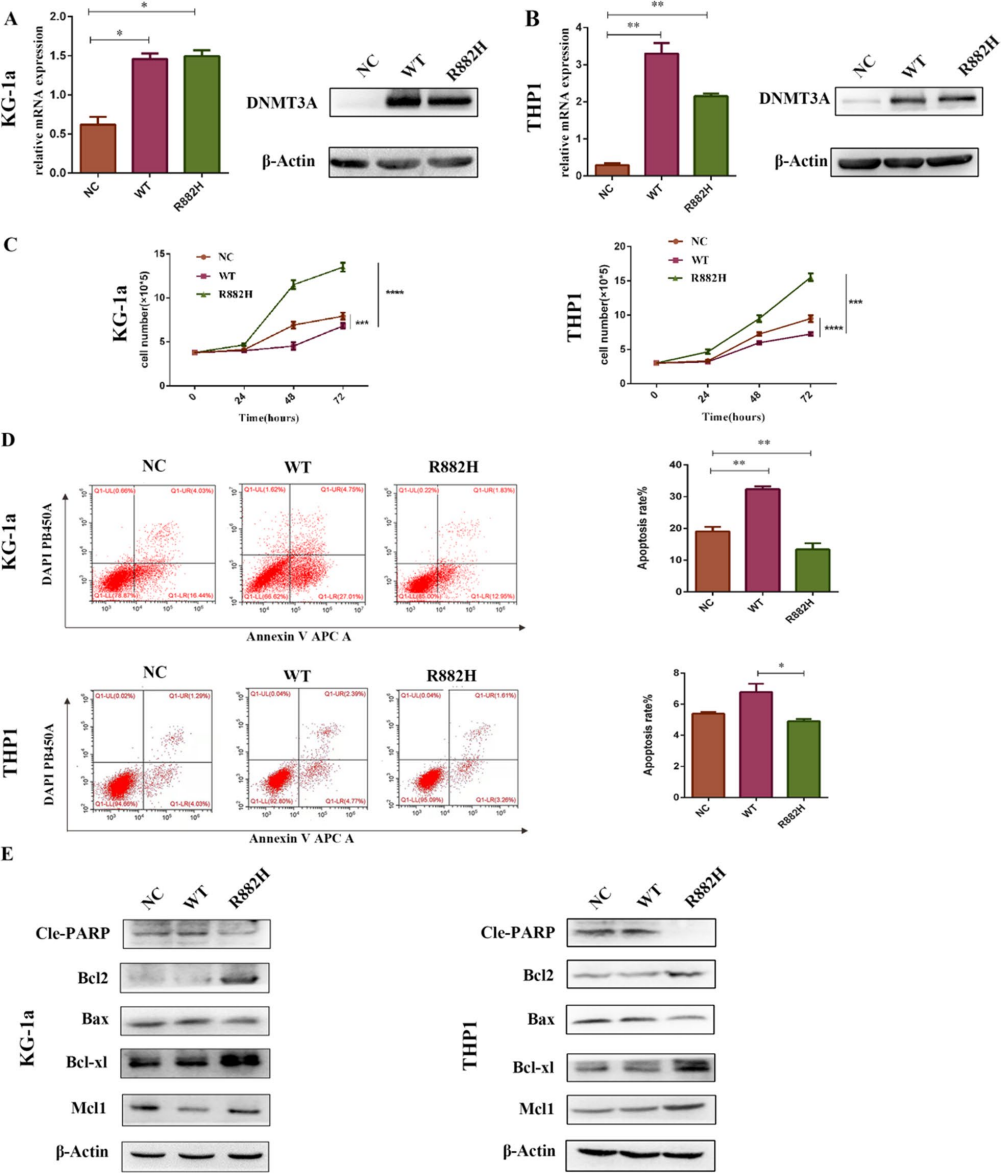
DNMT3A R882H mutation promotes cell proliferation and decreases cell apoptosis **A**, **B** The expression of DNMT3A in KG-1a and THP1 cells transduced with EV, DNMT3A-WT, and DMT3A-R882H was detected by qPCR and western blotting. **C** The growth curves of KG-1a and THP1 cells, transduced with EV, DNMT3A-WT and R882H. **D** The rate of cell apoptosis was detected by flow cytometry in KG-1a and THP1 cells stably transduced with EV, DNMT3A-WT and R882H in two cell lines. **E** Apoptosis related protein levels were examined by western blotting

To explore the role of DNMT3A R882H mutation in AML cells, we generated DNMT3A R882H mutant, wild-type (WT) and empty vector (EV) in KG-1a and THP1 cell lines (Fig. 1A, B). Cell counting results showed that cell proliferation was significantly enhanced in cells with DNMT3A-R882H mutant compared with that of EV and WT cells (Fig. 1C). Moreover, flow cytometry results suggested that the cells carrying the DNMT3A-R882H mutation have a reduced apoptosis rate compared with that of EV or DNMT3A-WT expressing cells (Fig. 1D). In addition, western blot results demonstrated that the presence of DNMT3A-R882H mutation can decrease the expression of pro-apoptotic proteins (cl-PARP, Bax) while increase the expression of anti-apoptotic proteins (Bcl2, Bcl-xl, Mcl1) (Fig. 1E).

### DNMT3A R882H mutation is associated with poor sensitivity to daunorubicin

**Fig. 2:**
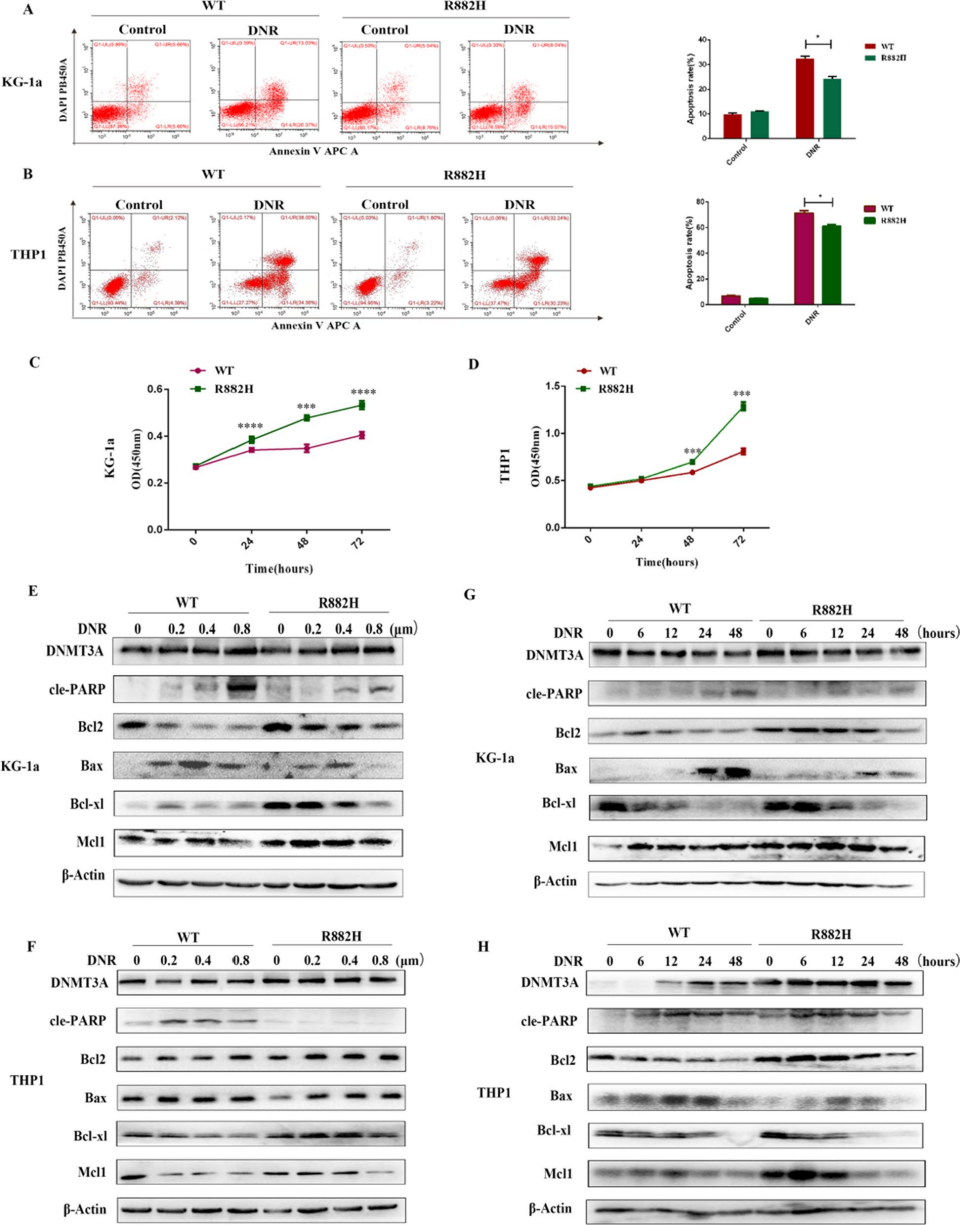
DNMT3A R882H mutation confers resistance to daunorubicin. **A**, **B** KG-1a and THP1 cells stably expressing DNMT3A-WT and the mutant were exposed to DMSO and DNR (0.4 μM) for 36 h and used for apoptosis analysis. **C**, **D** Sensitivity to DNR in KG-1a and THP1 cells expressing DNMT3A-WT and the mutant **E**, **F** Analysis of apoptosis related proteins in KG-1a and THP1 cells expressing DNMT3A mut and wild-type and treated with different concentrations of daunorubicin for 36 h. **G**, **H** Analysis of apoptosis related proteins in KG-1a and THP1 cells expressing DNMT3A mut and wild-type at different time points (0, 6, 12, 24, 48 h) after daunorubicin (0.4 μM) treatment

To better explore whether chemoresistance to daunorubicin is mediated by DNMT3A R882H mutation, we detected cell apoptosis rate by flow cytometry, with and without daunorubicin treatment. We found that the mutant cells exhibited a lower apoptosis rate compared with that of WT-expressing cells (Fig. 2A, B). The CCK-8 data (Fig. 2C, D) showed that DNMT3A R882H mutation reduced sensitivity to daunorubicin. Moreover, DNMT3A R882H mutant strongly suppressed cleaved PARP and Bax in response to different concentrations of daunorubicin (0, 0.2, 0.4, and 0.8 μM). However, western blot results showed that the inhibition effects of daunorubicin on anti-apoptotic proteins (Bcl2, Bcl-xl, Mcl1) was not as effective in DNMT3A mutant cells compared with that in cells transduced with DNMT3A-WT (Fig. 2E, F). Also, the same results were observed in mutant cells after treatment with DNR (0.4 μM) and detectedat different times (Fig. 2G, H).

### The NRF2/NQO1 pathway is activated in DNMT3A-R882H mutant cells

**Fig. 3:**
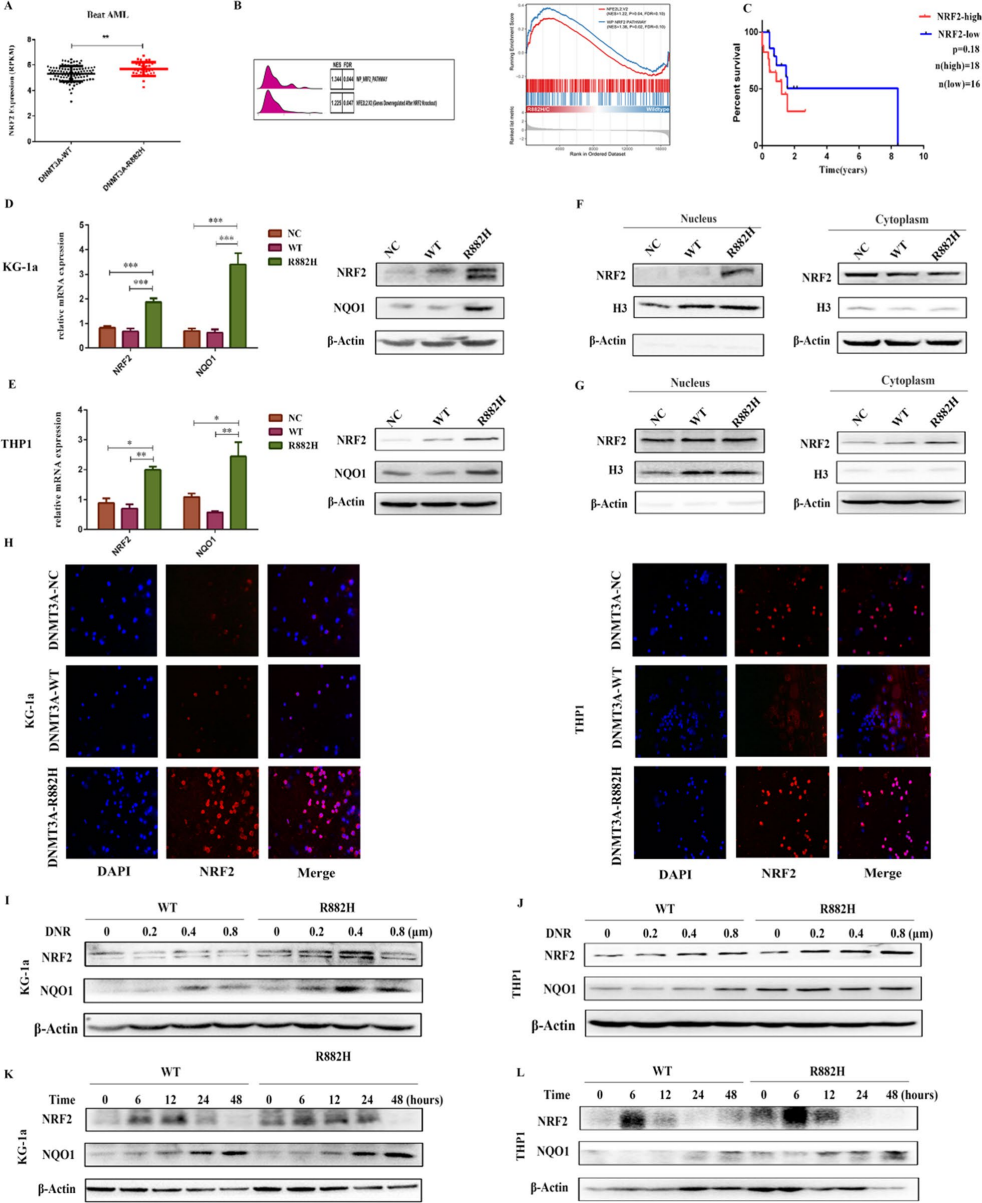
NRF2 is significantly augmented in AML cells expressing the DNMT3A R882H mutation. **A** NRF2 mRNA expression in samples of AML with the DNMT3A-R882 mutation compared with that in samples of AML with DNMT3A -WT. **B** GSEA analysis was used to detect NRF2 pathway enrichment in AML patients with the DNMT3A R882 mutation **C** The Beat AML database was used to analyze survival rate based on NRF2 expression. **D**, **E** The expression of NRF2 and NQO1 mRNA expression in WT and mutant KG-1a and THP1 cells were detected by qPCR and western blotting. **F**, **G** The expression of NRF2 in the nucleus and cytoplasm of KG-1a and THP1 cells expressing NC, WT and R882H was evaluated by western blot. **H** Cytoplasmic localization of NRF2 in KG1 and THP1 cells expressing DNMT3A WT and R882H. Immunostaining with anti-NRF2 (red) and DAPI (blue). **I**, **J** Immunoblot of NRF2 and NQO1 expressions in DNMT3A WT and mutant KG-1a and THP1 cells after treated with different concentrations of DNR for 24 h. **K**, **L** Immunoblot of NRF2 and NQO1 expressions in DNMT3A WT and mutant KG-1a cells and THP1 cells after treated with 0.4 μM DNR and harvested at different times

To explore whether NRF2 plays a role in AML of patients with the DNMT3A-R882 mutation, we detected the differential expression of NRF2 in AML patients with DNMT3A-WT and DNMT3A-mutation using the Beat AML database and found that the level of NRF2 was higher in AML patients with the DNMT3A R882 mutation (Fig. 3A). The results of Gene Set Enrichment Analysis (GSEA) also showed that the NRF2 pathway was enriched in DNMT3A R882 mutant group of AML patients (Fig. 3B). Kaplan–Meier analysis showed that patients with the DNMT3A-R882 mutation and high NRF2 expression have a shorter overall survival (Fig. 3C). To further determine whether DNMT3A R882H can regulate the expression of NRF2 in AML cells, NRF2 mRNA and protein expressions and its downstream target NQO1 were detected. The results (Fig. 3D, E) demonstrated that NRF2 and NQO1 expressions increased in mutant cells compared with those of WT and EV cells. NRF2 usually translocates into the nucleus and regulate oxidative stress related genes and detoxifying enzymes, and therefore, we also detected the expression of NRF2 in the nucleus. We observed that nuclear NRF2 had a higher expression in KG-1a mutant cells (Fig. 3F, G). The results of immunofluorescence also suggested that the expression of NRF2 was increased in mutant cells compared with that in DNMT3A-NC and DNMT3A-WT cells (Fig. 3H). In addition, we found that with the increased concentration of daunorubicin, the expression of NRF2 and NQO1 gradually increased in KG-1a and THP1 cells expressing the DNMT3A R882H mutant and the increment in mutant cells was much higher than that in cells transduced with the WT (Fig. 3I, J). Similarly, the increase rates of NRF2 and NQO1 were higher in mutant cells after treatment with DNR (0.4 μM) at different times (Fig. 3K, L).

### DNMT3A R882H mutation regulates the expression of NRF2 in a DNA-methylation-independent way

**Fig. 4:**
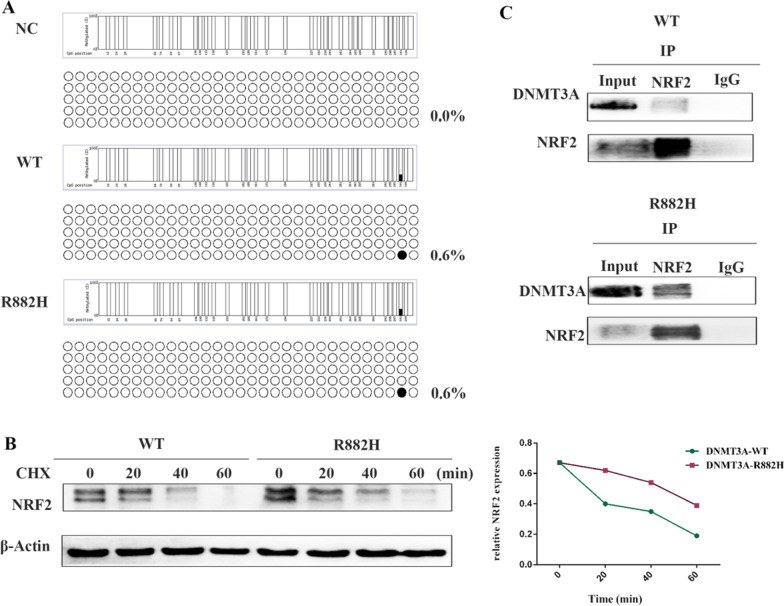
DNMT3A R882H mutation regulated the stability of NRF2. **A** Bisulfite sequencing analysis of the promoter-related CPG island of NRF2 in KG-1a cells expressing DNMT3A NC, WT, and R882H. **B** KG-1a cells expressing DNMT3A WT and DNMT3A R882H were incubated with CHX (10 μg/mL) at indicated times and the expression of NRF2 was detected by western blotting. **C** Total proteins extracted from KG-1a cells (NC, WT, R882H) were immunoprecipitated with anti-NRF2 antibody and immunoblotted with anti-DNMT3A and anti-NRF2 antibodies

To identify the mechanism by how the DNMT3A R882H mutation regulates the expression of NRF2, we detected the methylation level in the CPG islands of the NRF2 promoter in KG-1a cells expressing DNMT3A NC, WT, and R882H. Nevertheless, we observed no significant difference in methylation level of these cells (Fig. 4A). Then we treated KG-1a WT and mutant cells with CHX to investigate whether DNMT3A R882H mutation regulates NRF2 by influencing protein stability. Our data proved that DNMT3A R882H mutation reduced NRF2 degradation by increasing the half-life of NRF2 (Fig. 4B). Furthermore, the IP results showed that DNMT3A interacted with NRF2 in both WT and R882H cells (Fig. 4C).

### NRF2 inhibition increases the antitumor effect of daunorubicin in DNMT3A R882H mutant cells

**Fig. 5:**
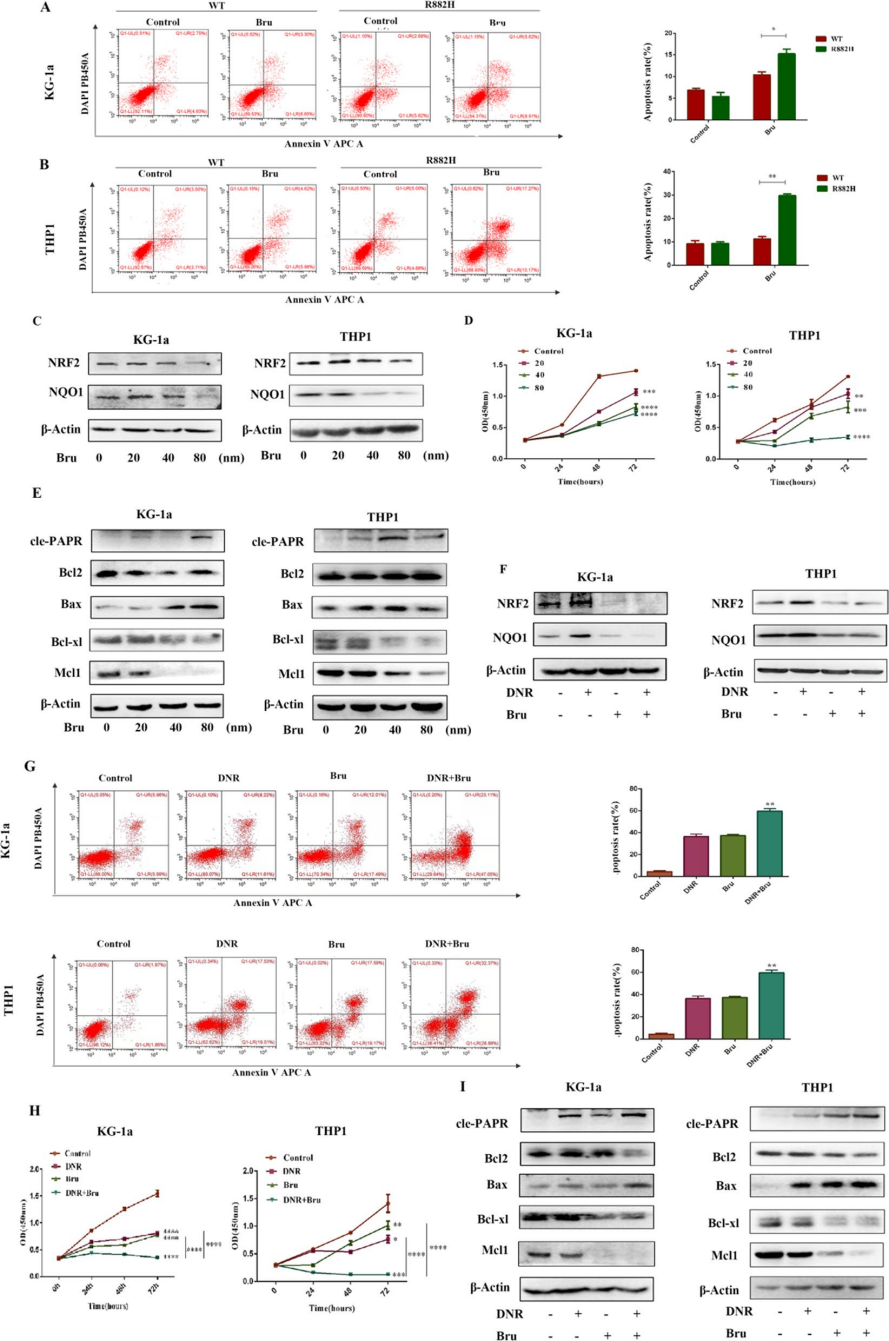
Inhibition of NRF2 improves sensitivity to DNR in DNMT3A R882H mutant cells. **A**, **B** Apoptosis rate was measured by flow cytometry in KG-1a and THP1 cells expressing WT and R882H after treatment with 40 nM brusatol. **C–E** Mutant KG-1a and THP1 cells were exposed to different concentrations of brusatol for 24 h to detect the expressions of NRF2, NQO1 and apoptosis-related proteins. **F** The expressions of NRF2, NQO1 and apoptosis related proteins were detected by Western blotting after treatment of KG-1a and THP1 mutant cells with DNR and brusatol. **G**–**I** The role of brusatol or DNR-brusatol combination on cell viability and cell apoptosis in KG-1a and THP1 mutant cells

To investigate the role of NRF2 in mutant cells, brusatol (Bru), a unique inhibitor of Nrf2 pathway, was used to treat cells expressing DNMT3A R882H mutation. Interestingly, DNMT3A-R882H mutant cells showed a higher apoptosis rate after brusatol treatment compared with that in DNMT3A WT cells, (Fig. 5A, B). We also found that brusatol can suppress the expression of NRF2 and NQO1 in DNMT3A-mutant cells (Fig. 5C). Furthermore, the treatment of brusatol inhibited cell proliferation and markedly triggered cell apoptosis in mutant cells (Fig. 5D, E). After treatment with DNR (0.4 μM) and Bru (40 nM), the NRF2/NQO1 pathway was significantly inhibited (Fig. 5F). Cell viability was also significantly decreased in the combined treatment group compared with that of DNR or Bru treatment alone group (Fig. 5G, H, J).

## Discussion

DNMT3A mutation happened in a variety of cancers such as colorectal cancer, lung cancer, chronic myelocytic leukemia, but is mainly with AML [32–34] and usually occur with other types of mutations, such as FLT3, NPM1, C/EBP alpha, resulting in synergistic effects in AML [35–37]. Being the most common mutation, DNMT3A R882H mutation can not only lower methylation enzymatic activity [38, 39] but can also co-act with other proteins to influence immune activity, cell apoptosis, proliferation, and DNA damage repair to regulate AML [40–42]. Researchers also found that Twist1, mTOR pathway, CDK1, and Dot1l [43–46] which might be the potential therapeutic targets of AML patients carry DNMT3A mutation. Reports have shown that the loss of DNMT3A or its mutant can cause an abnormal proliferation of hematopoietic stem cells, which then transform into preleukemic stem cells that are resistant to chemo-drugs and responsible for relapse in AML patients [47]. To better understand the role of DNMT3A R882H mutation in leukemia stem cells, the AML LSC-like cell line, KG1a, with the expression profile, CD34 + and CD38 − , was selected as our research objects [48]. Researchers have pointed out that AML patients with a DNMT3A mutation tend to be M4/M5 subtype [49], and therefore, we have also chosen the human acute monocytic leukemia cell line, THP1, for our studies. Our study confirmed that the DNMT3A R882H mutation did promoted cell growth and decreases cell apoptosis in mutant KG-1a and THP1 cells. DNR is an anthracycline drug that has two main mechanisms in cells, including inhibition of the nucleic acids synthesis and effects on the oxidoreductase system [50–52] which is effective in treating nonlymphocytic leukemia and acute lymphocytic leukemia [53–55]. Though DNR showed great effects in AML, toxicities associated with caused by daunorubicin, including cytopenias, hepatotoxicity, and cardiotoxicity [56–58] are major challenges. Our results identified that AML cells with a DNMT3A R882H mutation were not as sensitive to DNR as that of DNMT3A WT cells, which was in line with previous studies that confirmed that a high-dose daunorubicin improves the outcome of AML with a DNMT3A mutation [12, 13, 59].Then exploring how DNMT3A R882H mutation influence AML cells’ sensitivity to DNR is of great importance to improve the prognosis of AML patients after DNR treatment.

NRF2, first identified in 1994, is an important transcription factor which plays a role in cellular stability [60]. Studies have confirmed that the excessive activation of NRF2 can protect cells from apoptosis mediated by drug resistance in different cancers such as ovarian cancer, multiple myeloma, NSCLC tumors, colorectal cancer, and neck cancer [61–65]. Rabindranath Bera et al. pointed out that cells with a DNMT3A R882 mutation have a lower level of ROS when compared with that in WT cells, suggesting a potential role of DNMT3A R882H mutation in the regulation of the redox system in cells [42]. Therefore, we supposed that NRF2 may play a role in DNMT3A R882H related DNR-resistance. Researchers confirmed that NQO1 is related to anthracyclines metabolism [66, 67], and thus, we also investigated the role of NQO1 in our study. We found that NRF2 and NQO1 were increased in both in KG-1a and THP1 DNMT3A R882H mutant cells. We also observed that the expression of nuclear NRF2 was increased in KG-1a mutant cells. However, we did not notice similar results in THP1 cells, which may be explained by cell heterogeneity. Moreover, it was surprising to find that NRF2 expression was also increased in mutant cells after treatment with different concentrations of DNR, suggesting that DNR can also activate NRF2 and NQO1 in mutant cells. This increase may also explain the insensitivity of AML cells with DNMT3A R882H mutation to DNR. Given that NRF2 may help DNMT3A R882H mutant cells show low sensitivity to DNR, we treated mutant KG-1a and THP1 cell with brusatol, a specific inhibitor of NRF2. We observed that mutant cells were more sensitive to brusatol treatment, suggesting that the cells with the DNMT3A R882H mutation may be dependent on NRF2. Then we identified that brusatol did inhibit the NRF2/NQO1 pathway and decreased cell growth and promoted cell apoptosis. Moreover, the combination of DNR and brusatol not only reduced the expression of NRF2 and NQO1 but also dramatically promoted cell apoptosis. The great effects of this combination meant that NRF2 inhibition may help improve mutant cells’ sensitivity to DNR. Given that DNMT3A may regulate gene expression by influencing CpG methylation status of promoter regions, we detected the methylation of CpG islands of NRF2 promoter. However, the methylation status was not significantly different between DNMT3A-NC, DNMT3A-WT, and DNMT3A-R882H cells, suggesting that DNMT3A R882H may regulate NRF2 in a DNA methylation-independent way. Then we found that the stability of NRF2 increases in DNMT3A R882H mutant cells which indicated that there may be a posttranslational modification in DNMT3A R882H mutant cells that influences the expression of NRF2.

A study reported that AML patients with DNMT3A R882 mutation were resistant to venetoclax, a specific inhibitor of Bcl-2 [68]. Researchers have confirmed that the drug resistance to venetoclax is associated with the expression of Mcl-1 and Bcl-xl [69]. In our study, we found that AML cells with DNMT3A R882H had higher Bcl2, Bcl-xl and Mcl1 expressions compared with those in WT cells. In addition, the expression levels of the three proteins were higher in mutant cells compared with those in WT cells even when these cells were treated with DNR. These effects may also explain why AML patients with DNMT3A R882 mutation have a low sensitivity to venetoclax. Our research found that NRF2 inhibition can reduce the level of Bcl2, Bcl-xl, and Mcl-1, then in our future work, we will also explore whether NRF2 plays a role in venetoclax resistance to help improve the effects of venetoclax in AML patients with DNMT3A R882 mutation.

Certainly, our research has some limitations. Firstly, in the analysis of the survival rate of AML with DNMT3A R882 mutation, based on NRF2 expression, the number of AML cases in the database was too small to provide meaningful statistical results. We may collect a larger amount of clinical data to investigate whether NRF2 can work as a new biomarker to predict the prognosis of AML patients with DNMT3A R882 mutation in follow-on research. Secondly, we confirmed that DNMT3A R882H can influence the expression of NRF2 in a methylation-independent way, which suggests that DNMT3A R882H mutation not only influences DNA methylation of NRF2 promoter. Thirdly, we proved that the DNMT3A R882H mutation may regulate the stability of NRF2, and in our future work, we will investigate how DNMT3A R882H mutation affects the expression of NRF2 to better understand the role of DNMT3A R882H mutation. Furthermore, all experiments were carried in vitro which may not be enough to study how the DNMT3A R882H mutation mediates drug resistance to DNR via regulating NRF2. Thus, this phenomenon should be studied in vivo by generating DNMT3A R878H heterozygous mice models (a mice model mimics DNMT3A R882H in humans) [70] to study the relationship between DNMT3A R882H mutation and NRF2 and investigate the drug resistance to DNR.

**Fig. 6:**
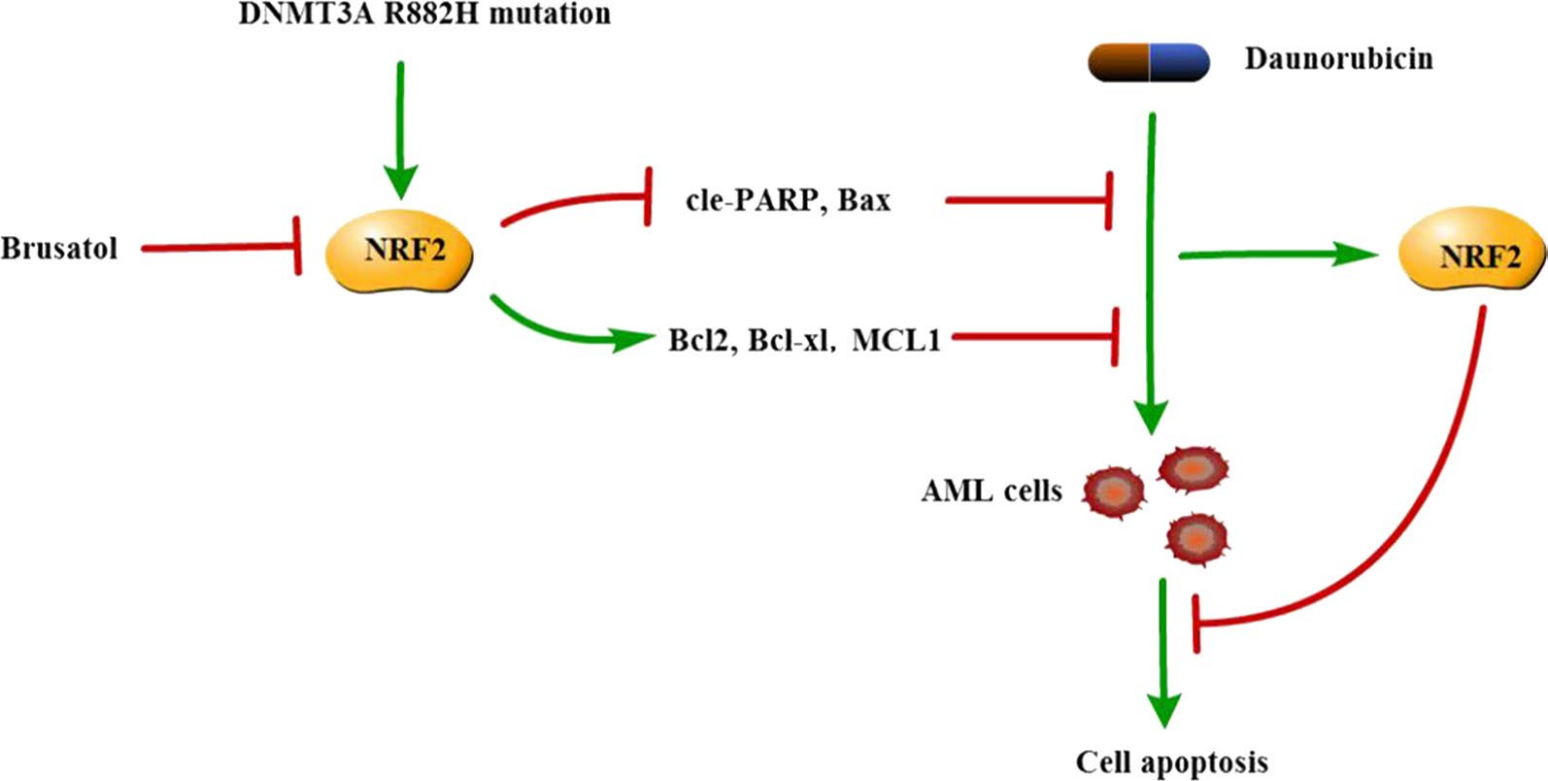
The mechanism of chemoresistance to DNR in AML cells with DNMT3A-R882H mutation

In summary, we find that NRF2 can be activated by the DNMT3A R882H mutation and that it mediates chemoresistance to DNR. Additionally, our study suggests that NRF2 can reduce mutant cells’ sensitivity to DNR by regulating cell proliferation and apoptosis. The mechanism is illustrated in the schematic diagram (Fig. 6). Targeting NRF2 can be helpful in making the DNR treatment more effective in AML patients with DNMT3A R882H mutation and minimizing side effects meanwhile.

## Materials and methods

### Cell lines and culture

KG-1a cell line was available in our own laboratory. The THP1 cell line was purchased from the cell bank of the Chinese Academy of Science (Kunming, China). The cells were cultured in RPMI-1640 medium supplemented with 10% FBS and then incubated at 37 °C under 5% CO_2_ atmosphere.

## Reagents

Daunorubicin (HY-15648B), the cell counting kit-8 (HY-K0301), Brusatol (HY-19543), Cycloheximide (HY-12320), 2-deoxy-d-glucose (HY-13966) and D-glucose (HY-B0389) were purchased from Med Chem Express (MCE, NJ, USA). RPMI-1640 medium was supplied by Gibco (MA, USA), fetal bovine serum (FBS; 900–108) was purchased from Gemini bio-products (Sacramento, CA, USA). The Nuclear and cytoplasmic protein extraction Kit (P0027) was obtained from Beyotime Biotechnology (China).

### Generation of DNMT3A R882H mutant cell lines

For the study, we generated KG-1a and THP1 DNMT3A R882H mutant cell lines. Briefly, lentiviral vectors expressing wild-type DNMT3A (DNMT3A -WT) and DNMT3A R882H (DNMT3A-mutant) were purchased from Genechem. An amount of 5 × 10^4^ leukemic cells per well were plated into a 24-well plate and infected with the above lentiviruses for 3 days using HitransG (for KG-1a cells) and HistransP (for THP1 cells) (Genechem). The stable expressing cell lines were selected with puromycin.

### Cell viability assay

The proliferation rates of cells were monitored using the CCK-8 assay kit. Briefly, the cells were seeded in 96-well culture plates at a density of 5 × 10^3^ cells/well and subsequently treated with indicated reagents. Finally, 10 μl of CCK-8 solution was added into the cell culture medium. Optical density (OD) measurements were carried out after incubation for 2 h. The OD value of each well was recorded at 450 nm using a micro-plate absorbance reader.

### Cellular apoptosis detection

For the evaluation of cellular apoptosis, the cells were seeded into 6-well plates at a density of 5 × 10^5^ cells /well and treated with indicated reagents. The apoptosis rate was determined by the Annexin V-FITC Apoptosis Detection Kit (SUNGENE BIOTECH, Tianjing). The flow cytometric analysis was performed using a CytoFLEX flow cytometer (Beckman Coulter, USA).

### Antibodies and western blot analysis

After indicated treatments, total protein, nuclear and cytoplasm proteins were extracted according to the instructions and the concentration of total protein was determined. The protein extracts were mixed with 5 × loading buffer, heated for 5 min, separated with sodium dodecyl sulfate poly acrylamide gel electrophoresis, and then transferred onto polyvinylidene fluoride membranes. After blocking with a 5% skimmed milk solution, the membranes were incubated with primary antibodies at 4 °C overnight. The next day, the membranes were incubated with secondary antibodies for 1 h at room temperature and then detected with an enhanced chemiluminescence (ECL) Ultra Western HRP Substrate kit (WBULS0100; EMD Millipore, USA) using an ECL visualization system (GE Healthcare, USA). The following antibodies were used for immunoblotting: β-Actin (TA-09) and H3 (bsm-33042 M) were purchased from ORIGENE (Beijing, China) and Bioss (Beijing, China), respectively. DNMT3A (ab188470) was obtained from abcam(Cambridge, MA, USA). NRF2 (16396–1-AP), MCL1 (16225–1-AP), Bcl2 (12789–1-AP), and Cle-PARP (13371–1-AP) were obtained from proteintech group (Rosemont, IL, USA). Bax (A5131), Bcl-xl (A5091), and NQO1 (A5464) were obtained from bimake. IgG for CO-IP was purchased from Cell Signaling Technology (USA).

### Immunofluorescence analysis

The cells were collected and washed with cold PBS, blocked in fetal sheep serum (Zhong-Shan Golden Bridge Biotechnology, China) for 30 min, and then incubated with the anti-NRF2 antibody at 4 °C overnight. The next day, the cells were washed with cold PBS and then incubated for 1 h with coralite 594-conjugated goat anti-rabbit secondary antibody (SA00013-4, Proteintech group), and then counterstained with DAPI (Beyotime) for 5 min. Images of the cells were analyzed by inverted fluorescence microscopy. The exposure time for DAPI and red fluorescence were both 150 ms.

### Bisulfite sequencing

Genomic DNA samples were isolated from KG-1a ells, that were stably transduced with DNMT3A-NC, DNMT3A-WT, and DNMT3A-R882H mutant, and subjected to bisulfite conversion with the EZ DNA methylation-gold kit (Zymo Research, Beijing) according to the manufacturer's instructions. Bisulfite-converted DNA was amplified by PCR using specific primers. The PCR products were cloned using the Pmd18-T vector (Takara, Japan) and the CpG methylation pattern was visualized after bisulfite sequencing. The sequencing primers used for the bisulfite sequencing analysis of the NRF2 gene were forward primer: TAATTTTAAATTAGGGAGGYGTAGT and reverse primer: ACAAAAATCCRAACCCTTCC.

### Co-IP

Protein A/G Magnetic Beads (MCE), and antibodies (NRF2 and IgG) were added into two tubes respectively, and incubated on an inverted mixer for 2 h to mix the beads with the antibodies. After washing with PBST, the protein extracts were incubated with the beads on an inverted mixer at 4 °C overnight. Next day, the proteins were analyzed with western blotting.

### Cycloheximide assay

To investigate protein stability of NRF2 in DNMT3A-WT and DNMT3A-R882H KG-1a cells, these later were treated with 10 μg/mL cycloheximide (CHX; MCE) to block de novo protein synthesis for 0, 20, 40 and 60 min. Then the expression of NRF2 was analyzed by western blot.

### Quantitative real-time PCR

PrimeScript ™ RT reagent Kit (Takara, Japan) was used to synthesize cDNA. qRT-PCR was performed on a CFX Connect ™ real-time PCR operating system (Bio-Rad, USA) using the SYBR ® Premix ExTaq ™ II kit (Takara, Japan). The primers were synthesized at Sangon Biotech (Shanghai, China). The sequences of all primers used for qRT-PCR are shown in Table 1.

**Table 1:** Sequences of all primers used of quantitative real-time PCR

Gene	Forward/reverse	Primer Sequence (5′–3′)
DNMT3A	F	CATCTGCATCTCCTGTGGG
	R	GCAGTTTTGGCACATTCCTC
NRF2	F	TCAGCGACGGAAAGAGTATGA
	R	CCACTGGTTTCTGACTGGATGT
NQO1	F	GAAGAGCACTGATCGTACTGGC
	R	GGATACTGAAAGTTCGCAGGG
β-Actin	F	TGACGTGGACATCCGCAAAG
	R	CTGGAAGGTGGACAGCGAGG

### Statistical analysis

The Beat AML database was used to identify the expression of NRF2 in DNMT3A-WT and AML patients with DNMT3A-R882 mutation (www.vizome.org) [32]. For Gene Set Enrichment Analysis (GSEA), the data was also obtained from the Beat AML database. The overall survival of AML patients with DNMT3A-R882H mutation was divided into high and low NRF2 expression groups using the Beat AML dataset (n = 42) and evaluated by Cox proportional hazards regression models. The p-value was calculated by the log-rank test and corrected according to the database description.

The statistical difference between the two groups was compared using unpaired Student’s t test. The data were presented as mean ± standard deviation (SD). *p*-value < 0.05 was considered statistically significant. “*”,“**”, “***” and “****”shown a statistical significance with *p*-value < 0.05, < 0.01, < 0.001, < 0.0001, respectively. All statistical analyses were carried out using GraphPad Prism 5.0 and the SPSS Statistics software (19.0).

## Data Availability

Data and materials will be shared.

## Abbreviations

DNMT3A: DNA methyltransferase 3A
AML: Acute myeloid leukemia
NRF2: Nuclearfactor-E2-related factor
DNMT3A-WT: DNMT3A-wild type
DNMT3A-R882H: DNMT3A -Arg882His
NQO1: NAD (P) H: quinone oxidoreductase 1
Dau: Daunorubicin
Bru: Brusatol
CHX: Cycloheximide
